# Dynamic capsule restructuring by the main pneumococcal autolysin LytA in response to the epithelium

**DOI:** 10.1038/ncomms10859

**Published:** 2016-02-29

**Authors:** Colin C. Kietzman, Geli Gao, Beth Mann, Lance Myers, Elaine I. Tuomanen

**Affiliations:** 1 Department of Infectious Diseases, St Jude Children's Research Hospital, Memphis, Tennessee 38103, USA

## Abstract

Bacterial pathogens produce complex carbohydrate capsules to protect against bactericidal immune molecules. Paradoxically, the pneumococcal capsule sensitizes the bacterium to antimicrobial peptides found on epithelial surfaces. Here we show that upon interaction with antimicrobial peptides, encapsulated pneumococci survive by removing capsule from the cell surface within minutes in a process dependent on the suicidal amidase autolysin LytA. In contrast to classical bacterial autolysis, during capsule shedding, LytA promotes bacterial survival and is dispersed circumferentially around the cell. However, both autolysis and capsule shedding depend on the cell wall hydrolytic activity of LytA. Capsule shedding drastically increases invasion of epithelial cells and is the main pathway by which pneumococci reduce surface bound capsule during early acute lung infection of mice. The previously unrecognized role of LytA in removing capsule to combat antimicrobial peptides may explain why nearly all clinical isolates of pneumococci conserve this enzyme despite the lethal selective pressure of antibiotics.

## Abstract (web summary)

Pneumococci produce a carbohydrate capsule that protects them against components of the host immune system but sensitizes them to host antimicrobial peptides. Here, Kietzman *et al.* show that pneumococci respond to antimicrobial peptides by capsule shedding, which requires the main autolysin LytA.

## Graphical abstract

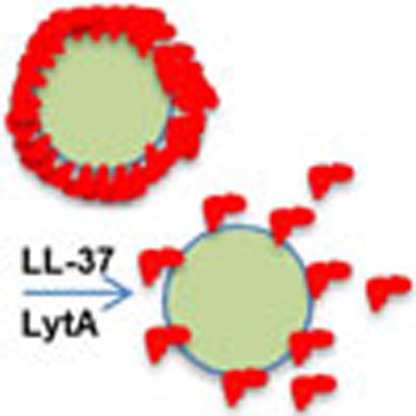

Bacterial pathogens produce complex carbohydrate capsules that dominate surface chemistry, inhibit phagocytosis and induce immunological responses by the host. The pneumococcus, or *Streptococcus pneumoniae*, produces over 90 chemically distinct capsule types and the inability to produce capsule renders the bacterium severely attenuated. As with most Gram-positive pathogens, the pneumococcal capsule is thought to be covalently linked to the peptidoglycan of the cell wall in most strains, though the specific nature of the attachment has remained elusive^1^; however, it is clear that in different host tissues, such as epithelial surfaces, the pneumococcus can rapidly reduce encapsulation through unknown mechanisms^2^. Reduced encapsulation promotes intimate adherence to host cells and increases the ability to invade epithelial and endothelial barriers, a prerequisite of systemic disease^3^. Surprisingly nothing is known about how the pneumococcus removes capsule from its surface once it is covalently attached. We hypothesized that tissue-specific signals in the host direct the pneumococcus to modulate the amount of surface attached capsule through activation of a removal pathway.

Immediately upon interaction with colonizing or infecting organisms, the respiratory epithelium controls microbial populations through several innate defenses. Amongst these, cationic antimicrobial peptides (CAMPs) are one of a relative few epithelial surface molecules with direct microbicidal activity. In humans, airway epithelia produce a limited spectrum of CAMPs, mainly members of the β-defensins and the cathelcidin-derived LL-37 (refs 4, 5, 6). Despite a paucity of studies, data suggests that in bronchoalveolar lavage (BAL) fluid LL-37 is the most abundant CAMP, compared with β-defensins 1 and 2, that are also present in detectable amounts^7^. Recent studies have confirmed that LL-37 plays a role in defense against respiratory pathogens, including both viral and bacterial infections^6^,^8^,^9^,^10^. In the specific case of the pneumococcus, CAMPs play a crucial role in bactericidal control of pneumococcal infections. Within high-performance liquid chromatography-separated fractions of human neutrophils, pneumococcal killing was observed mainly in fractions containing the CAMPs human neutrophil proteins 1–3 (ref. 11). Further it has been shown that adding purified pneumococcal capsule can protect unrelated, unencapsulated bacteria from the bactericidal effects of human CAMPs presumably by acting as a scavenger of unbound peptides^12^.

While purified pneumococcal capsule is thought to act as a decoy for CAMPs, the production of surface-associated capsule has been found to adversely affect survival in response to CAMPs. Genetic inactivation of capsule production in several serotypes rendered pneumococci more sensitive to killing by CAMPs^11^. Capsule therefore has dual roles in the response of pneumococci to CAMPs, sensitizing those strains that are making capsule to bactericidal effects, while purified capsule has the ability to protect unencapsulated strains from unbound antimicrobial peptide in the environment. Thus we hypothesized that CAMPs were an important molecule involved in the modulation of encapsulation of pneumococci. Indeed, we show here that exposure to CAMPs leads to the accumulation of pneumococcal capsule in the supernatant of cultures, and that growth in a CAMP-rich environment can lead to almost complete loss of surface capsule. This shedding of capsule from the pellet fraction to the supernatant in response to CAMPs is dependent upon the pneumococcal autolysin LytA, and occurs during acute lung infection in both a CAMP- and LytA-dependent manner. Our results reveal a novel pathway for removal of surface-associated capsule that is both protective against the bactericidal effects of CAMPs and provides the first molecular mechanism for the loss of capsule observed when bacteria contact epithelial surfaces.

## Results

### Capsule production and sensitivity to LL-37

**Figure 1:**
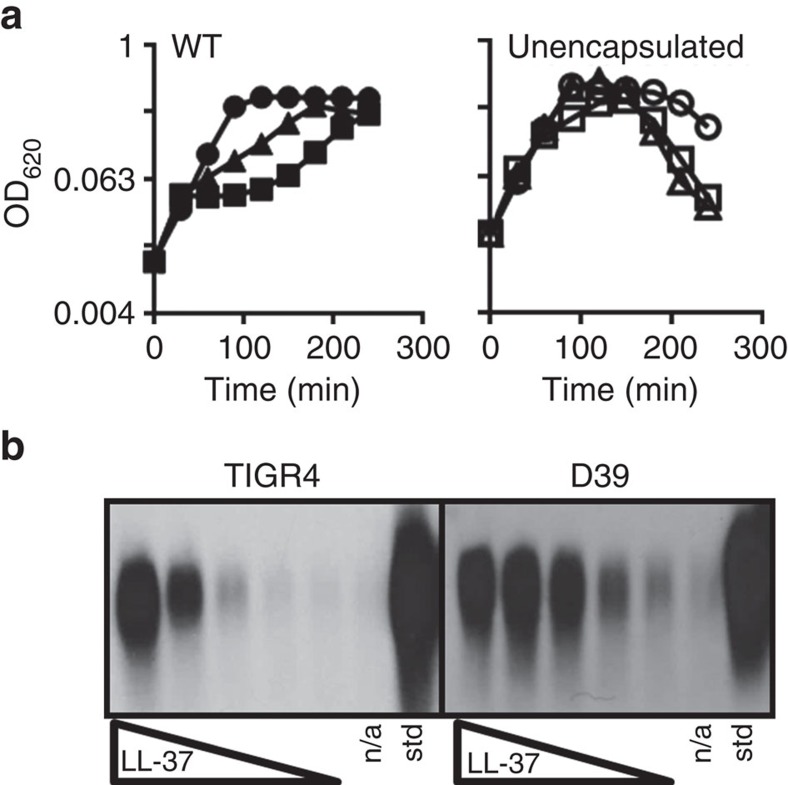
Effects of LL-37 on viability and capsule shedding. (**a**) Representative growth curve of WT strain TIGR4 (left) or a TIGR4-derived mutant in the capsule synthesis genes *cps4E-F* (right) grown in C+Y. LL-37 was added at *t*=0 at 16 μg ml^−1^ (triangles), 32 μg ml^−1^ (squares) or was omitted from cultures (circles), and growth at 37 °C was monitored by optical density. (**b**) WT strains TIGR4 or D39 were treated with LL-37 at concentrations ranging from 0.25 to 4 μg ml^−1^ in a standard capsule shedding assay (see Methods). The resulting supernatants were then processed and analysed via capsule blot (see Methods). n/a: no addition; std: purified capsule. Experiments were repeated at least three times.

To begin to understand the relationship between capsule and antimicrobial peptides, we studied the growth of encapsulated and unencapsulated pneumococci in the presence of LL-37. We found, similar to a previous report^11^, that growth of encapsulated bacteria was inhibited by LL-37 at 16 and 32 μg ml^−1^, whereas a mutant producing no capsule was not inhibited at these concentrations (Fig. 1a). This observation raised the question of whether pneumococci dynamically alter encapsulation to improve survival when in contact with CAMPs. When we examined the capsule after pneumococci were treated with LL-37, we found capsule increased in the supernatant in response to LL-37 in a dose-dependent manner (Fig. 1b). This response was evident in two commonly utilized strains, TIGR4, which was isolated from a meningitis case and produces a serotype 4 capsule, and D39, which is a bacteremia serotype 2 isolate (although the response of D39 appears to saturate or level off at lower LL37 concentrations than for TIGR4; Fig. 1b)^13^. Together, reduced growth by encapsulated strains and accumulation of capsule in the supernatant after LL-37 exposure suggested that capsule shedding was an active response to antimicrobial peptides in order to reduce the amount of surface-associated capsule.

The relationship between pneumococcal capsule and protection of unrelated unencapsulated bacterial from CAMP-mediated killing suggested that LL-37 may bind primarily to capsule in encapsulated strains. Using fluorescently labelled LL-37, the membrane selective dye Mitotracker Green FM, and anti-capsule antiserum, we observed that LL-37 primarily co-localized to the bacterial membrane, as has been reported for the majority of CAMPs (Supplementary Fig. 1a). The localization of the majority of labelled LL-37 at the membrane was also not dependent on capsule synthesis as co-localization of membrane stain and LL-37 was observed in the mutant lacking capsule (Supplementary Fig. 1b). The lack of co-localization between labelled LL-37 and capsule immuno-staining suggests that direct binding to capsule is either transient, perhaps as a transition to membrane binding, or, at steady state, occurs at levels significantly lower than binding to the bacterial membrane.

### Encapsulation decreases over time during growth in LL-37

**Figure 2:**
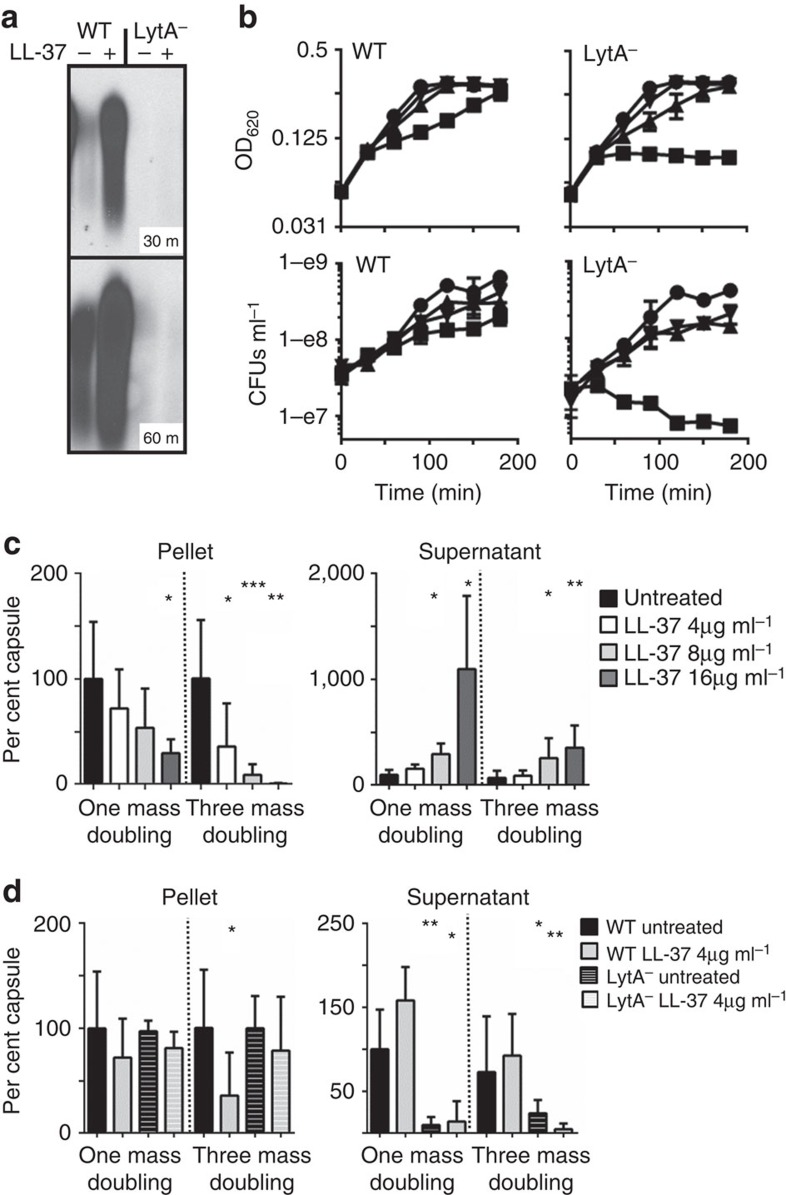
Role of LytA autolysin in capsule shedding. (**a**) WT TIGR4 or the isogenic deletion mutant in the *lytA* gene (LytA^−^) were treated with 4 μg ml^−1^ LL-37 in a capsule-shedding assay for 30 or 60 min as indicated. The supernatants were then analysed by capsule blot. (**b**) TIGR4 (WT) or the mutant in *lytA* (LytA^−^) were treated at *t*=0 with LL-37 at 16 μg ml^−1^ (squares), 8 μg ml^−1^ (triangles), 4 μg ml^−1^ (inverted triangles) or untreated (circles). Cultures were then monitored by optical density and samples collected for enumeration of viable CFUs at the indicated times. (**c**) TIGR4 (WT) was grown in C+Y to OD 0.4. Cultures were then back diluted to OD 0.2 (1 mass doubling) or OD 0.05 (three mass doublings) and LL-37 was added at the indicated concentrations. At OD 0.4 the cultures were harvested and pellet and supernatant fractions were analysed by capsule blot. Relative capsule amounts were calculated by comparison with a purified capsule standard and were normalized to untreated cells for each fraction. The results are the mean and s.d. from three independent experiments (100% pellet: 55.9 μg ml^−1^±32.1 *n*=3; supernatant 2.68 μg ml^−1^±0.827, *n*=3). (**d**) The LytA^−^ strain was grown as in **c** and treated with 4 μg ml^−1^ LL-37 where indicated. For reference, the results for WT in the same conditions in **c** were compared and plotted against the LytA^−^ results. *P<0.05 **P<0.01 ***P=0.001 unpaired *t*-test with Welch's correction.

To determine the rate and degree of shedding at physiological concentrations of LL-37, that have been measured up to 25 μg ml^−1^ in BAL fluid^7^,^14^, the amount of cell-associated and released capsule was measured in a dose- and time-dependent manner (Fig. 2c). At high concentrations of LL-37 (16 μg ml^−1^) after one mass doubling, 60% of capsule in the cell pellet (cell-associated) was lost compared with capsule in the untreated pellet, which suggests an active process of removal rather than simply cessation of capsule synthesis followed by dilution of remaining capsule to daughter cells. In support of this model, as cell-associated capsule decreased, there was a marked increase in the amount of capsule in the supernatant (Fig. 2c). After three mass doublings, wild-type (WT) pneumococci grown in 16 μg ml^−1^ LL-37 lost 95% of the cell-associated capsule compared with untreated cells (Fig. 2c). Therefore, growth over time in physiological concentrations of LL-37 can cause the pneumococcus to become nearly unencapsulated while releasing at least a portion of that capsule into the surrounding environment.

### The role of LytA in capsule shedding and protection from LL-37

Since the majority of capsule serotypes are attached to cell wall, we tested if mutants in predicted carbohydrate-active, cell wall localized or cell wall-active enzymes-mediated shedding^11^,^15^ (Supplementary Table 3). Of the mutants tested, only the major pneumococcal autolysin, the cell wall amidase LytA, was necessary for capsule release in response to LL-37 (Fig. 2a); enzymes linked to other autolytic processes, such as LytC, did not markedly affect shedding under these conditions. The LytA autolysin cleaves the amide bond between the peptide stem and *N*-acetyl muramic acid residues in peptidoglycan of cell wall. When activated by antibiotics such as penicillin or vancomycin, LytA cleaves cell wall to cause cell lysis in a process that, once triggered, leads irreversibly to death. Unlike LytA-dependent killing by antibiotics, we observed no lysis or bacterial death caused by LL-37 addition, even at 16 μg ml^−1^, which was several times higher than was sufficient to cause capsule shedding (Fig. 2b). Continued viability and even growth in LL-37 suggested that, unlike antibiotics, the activation of LytA by CAMPs and subsequent cell wall clipping is not fatal and is most likely actively controlled, or reversible. In the absence of functional LytA, where capsule was not shed, pneumococci became up to 2–4-fold more sensitive to LL-37, and indeed at the highest level tested lost 90% viability over 3 h (Fig. 2b). This shows for the first time that LytA is a CAMP resistance factor, and that LytA activation and subsequent loss of capsule in response to CAMPs is a survival mechanism for the pneumococcus. In support of this model, after growth in LL-37 for one or three mass doublings, the LytA mutant was unable to shed capsule, as indicated by absence of capsule from the supernatant, and it retained the majority of cell-associated capsule compared with WT cells grown in the same conditions (Fig. 2d). Localization of LL-37 to the bacterial membrane was also observed in the LytA mutant (Supplementary Fig. 1b).

### Complementation of LytA^−^ shedding defect

**Figure 3:**
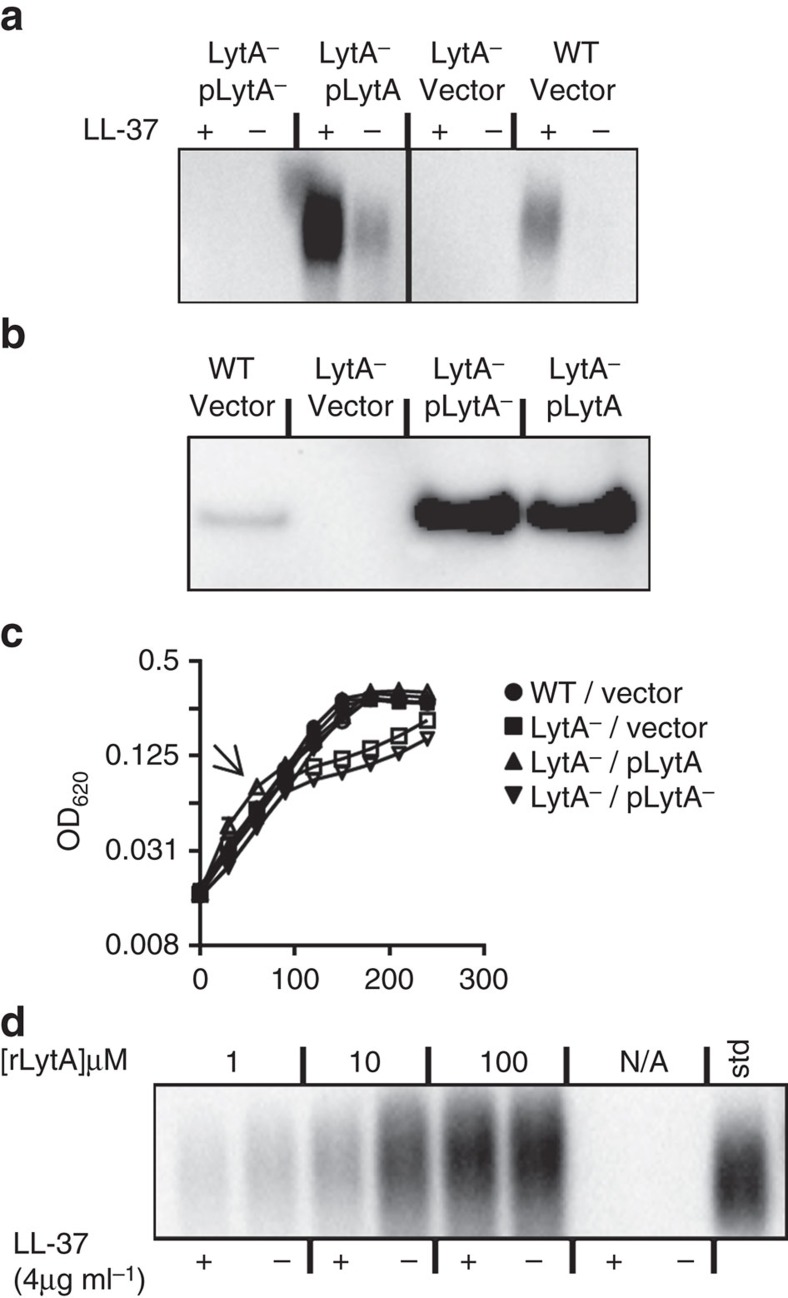
Amidase activity of LytA is required for capsule shedding. (**a**) TIGR4 (WT) with empty vector, the *lytA* mutant (LytA^−^) with empty vector or vector containing a WT *lytA* allele (pLytA) or an allele with mutations in active-site residues (pLytA^−^) were subjected to capsule shedding assay. The supernatant fractions were then analysed by capsule blot. (**b**) Cultures from **a** were harvested, mechanically lysed, and lysates, normalized for total protein, were analysed by western blot using LytA antiserum (see full blot in Supplementary Fig. 6a). (**c**) Representative growth curve of strains from **a** grown in C+Y. At the arrow LL-37 was added to indicated cultures (open symbols) at 8 μg ml^−1^. (**d**) The *lytA* mutant was subjected to a capsule-shedding assay, and recombinant LytA protein was added at the indicated concentration to the assay. LL-37 was added to the indicated cultures at 4 μg ml^−1^. The supernatant fraction was then analysed by capsule blot.

We sought to confirm that the amidase activity of LytA underlies capsule shedding. When expressed from a plasmid, WT LytA was able to complement a LytA null strain for capsule shedding in the presence of LL-37 (Fig. 3a). The complemented strain shed higher amounts of capsule than WT pneumococci probably because of multi-copy overexpression of LytA (Fig. 3a,b); however, shedding was still stimulated by the addition of LL-37 suggesting that endogenously overproduced LytA is still regulated similarly to LytA produced at normal protein levels. Despite similar levels of overexpression, a site-directed mutant allele of *lytA* with altered predicted active-site residues, was unable to complement the shedding defect of the LytA null strain, suggesting that LytA does not use another enzymatic activity beyond the amidase function to drive capsule shedding. Supporting a model where it is the enzymatic activity of LytA that is necessary for capsule shedding and capsule shedding is the main activity by which LytA acts as a CAMP resistance factor, resistance to LL-37 was restored by complementation of WT but not mutant LytA (Fig. 3c). When recombinant full-length LytA protein exogenously produced in *Escherichia coli* was added in shedding assays to the LytA^−^ strain, it was apparent that capsule was shed into the supernatant regardless of addition of LL-37 (Fig. 3d). Unlike endogenously produced LytA, this suggests that the normal regulatory functions evident in shedding assays where LytA is produced in the cell are unable to control LytA incorporated from the surrounding milieu. A recombinant LytA with the choline-binding domains genetically deleted failed to cause either cell lysis or shedding when added at the same concentrations as full-length LytA (Supplementary Fig. 2). The unregulated shedding of LytA added exogenously in the absence of the LL-37 trigger contrasts to LytA driven autolysis, where addition of purified LytA is insufficient to cause autolysis but requires either entry into stationary phase or inclusion of autolytic triggers such as antibiotics or detergents^16^. Together these findings suggested that factors other than enzymatic activity distinguished LytA physiology during autolysis versus shedding.

### Capsule shedding by LytA at distinct sites from autolytic LytA

**Figure 4:**
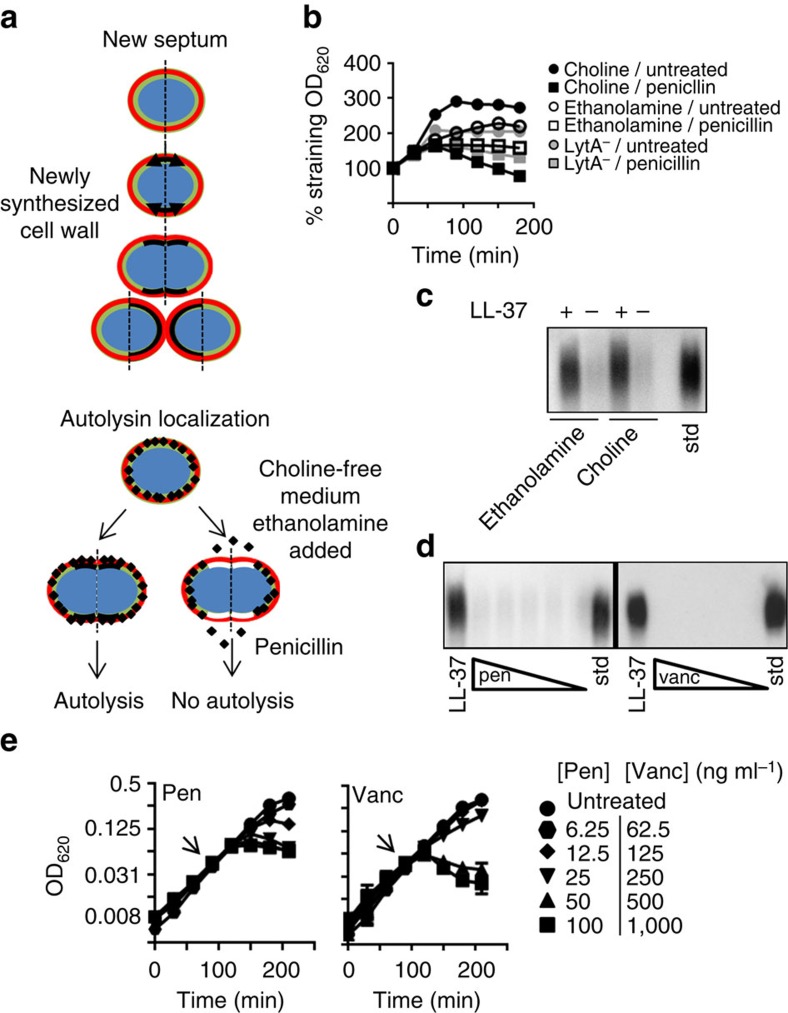
Distinction between LytA activation during capsule shedding versus by antibiotics. (**a**) Schematic representation of localization of cell wall synthesis and autolysin (red=capsule, green=old cell wall, black=new cell wall containing choline, white=new cell wall containing ethanolamine, LytA=black diamonds). (**b**) TIGR4 was grown as described for ethanolamine pulse experiments (Methods). Penicillin was added as indicated at 40 ng ml^−1^. For an autolytic negative reference the mutant in *lytA* (LytA^−^) grown in choline was included. (**c**) Cultures from **b** after ethanolamine or choline pulse were harvested and used in a standard capsule-shedding assay and the supernatant fractions were analysed by capsule blot. Std: purified capsule polysaccharides. (**d**) TIGR4 was subjected to a capsule-shedding assay with either LL-37 at 4 μg ml^−1^, pencillin (10–80 ng ml^−1^) or vancomycin (100–800 ng ml^−1^). Supernatants from these assays were then analysed by capsule blot. Std: purified capsule polysaccharides. (**e**) At the arrows, cultures of TIGR4 were exposed to either penicillin (Pen) or vancomycin (Vanc) at the indicated concentrations and growth was monitored by optical density.

One physiological function of LytA is to cause autolysis, a cell density-dependent phenomenon characteristic of major pathogens, such as pneumococcus, *Haemophilus* and meningococcus^17^,^18^. Importantly, this activity of LytA is triggered by β-lactam antibiotics and is the main mechanism by which pneumococci and other autolytic bacteria are killed^19^,^20^. Previous studies have shown LytA involved in autolysis is localized to the cell wall growth zone; however, secreted LytA is bound to choline on the cell wall and, thus is, likely distributed around the cell as teichoic acids migrate away from the septum during growth^21^. To test if shedding involved LytA at sites outside the cell wall growth zone, we utilized a unique facet of pneumococcal biology: the pneumococcus requires choline for cell wall synthesis and proteins such as LytA are anchored to cell wall by interaction with choline. When choline is removed from the growth medium and ethanolamine is added, the pneumococcus substitutes ethanolamine into teichoic acids in the geographically restricted growth zone^21^. The result of this ethanolamine pulse is newly synthesized cell wall with exiguous choline and high ethanolamine levels only at the septum/cell wall growth zone (Fig. 4a). Since LytA attaches to cell wall by binding to choline, these pulses result in a localized band of cell wall depleted of LytA-binding sites (Fig. 4a). Pneumococci pulsed with ethanolamine showed the expected failure of penicillin to activate LytA for autolysis, since LytA was reduced at the growth zone where penicillin-induced triggering occurs (Fig. 4b). However, capsule shedding occurred regardless of ethanolamine or choline treatment (Fig. 4c). This indicates that unlike autolysis, capsule shedding involves activation of LytA that is localized outside the growth zone. Consistent with this conclusion, when the autolysis-inducing cell wall antibiotics vancomycin and penicillin were used at lytic concentrations (Fig. 4e), no capsule shedding was observed (Fig. 4d). Combined, these results indicate that capsule shedding and autolysis are separate processes carried out by geographically distinct populations of LytA. Unlike capsule shedding, the regulatory functions controlling antibiotic-induced autolysis affect LytA present at the cell wall growth zone; from our experiments, activation of LytA through antimicrobial peptides involves enzyme in areas of cell wall spatially distinct from autolysis.

### Specificity of capsule shedding

**Figure 5:**
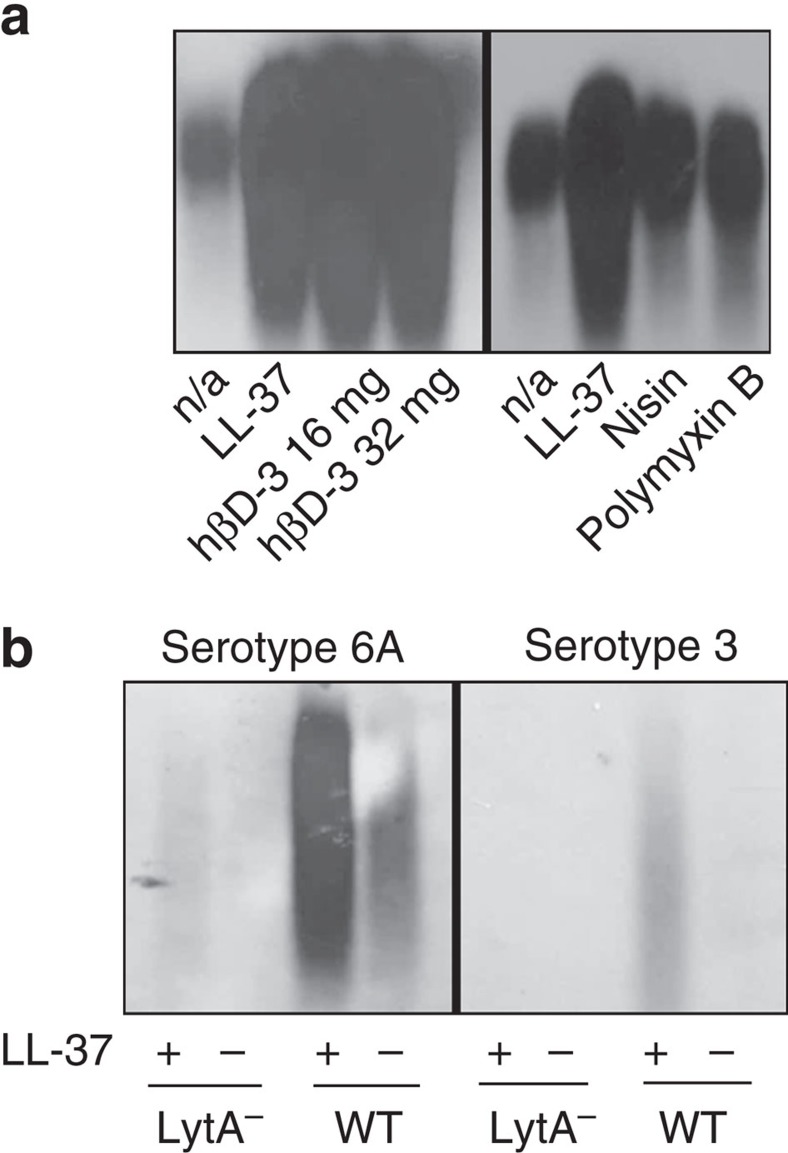
Capsule shedding as a function of antimicrobial peptides or capsule serotype. (**a**) TIGR4 was treated with LL-37 (4 μg ml^−1^), human β-defensin 3 (hβD-3) at the indicated concentration, nisin (64 μg ml^−1^) or polymyxin B (32 μg ml^−1^). The supernatants from these assays were then analysed by capsule blot. n/a: no addition. (**b**) Serotypes 6A and 3 strains were subjected to the shedding assay with LL-37 (4 μg ml^−1^) and the supernatants were analysed by capsule blot.

The above data illustrate that pneumococci shed capsule to improve survival when they encounter LL-37. Humans produce a limited number of CAMPs with no apparent homology save for overall positive charge^22^. However, like LL-37, exposure to the human CAMP β-defensin 3 stimulated robust capsule shedding at sub-bactericidal concentrations (Fig. 5a). Similarly exposure to the bacterially derived antimicrobial peptides nisin and polymyxin B resulted in increased capsule in the supernatant (Fig. 5a). Capsule shedding in response to multiple structurally unrelated CAMPs suggested that shedding was a response to a shared activity of CAMPs. Many CAMPs interact with the peptidoglycan synthesis intermediate lipid II, which contains undecaprenyl phosphate, a membrane anchor involved in both cell wall and capsule synthesis^23^,^24^,^25^,^26^. We hypothesized that pneumococci might shed capsule because of CAMP interaction with this shared intermediate. Serotype 3 pneumococcal capsule is unique in that it is synthesized using a phosphatidyl glycerol lipid and therefore shares no intermediate anchor molecules with cell wall synthesis^27^. However, when exposed to LL-37, a serotype 3 strain also displayed LytA-dependent capsule shedding, similar to the laboratory strains TIGR4 and D39 tested earlier, and a non-invasive serotype 6A isolate (Fig. 5b). Thus, it is unlikely that the signal inducing capsule shedding is sequestration of a capsular-undecaprenyl phosphate intermediate by antimicrobial peptides. It remains to be determined how shedding relates to membrane poration, the main bactericidal mechanism of CAMPs.

In addition to utilizing an alternate phospholipid for it's synthesis, serotype 3 capsule is notable in that it is thought to be surface-associated in a non-covalent manner^1^. The finding that a serotype 3 strain shed capsule suggested that rather than recognizing a unique capsule/cell wall attachment, capsule shedding might involve the release of multiple cell wall-associated molecules. Indeed probing for a teichoic acid-attached protein and peptidoglycan sorted protein (CbpA and pilus, respectively) indicated that these molecules increased in the supernatant in a LytA- and LL-37-dependent manner (Supplementary Fig. 3). The presence of proteins attached to alternate sites in the cell wall in the supernatant after the shedding response suggests that the LytA response to CAMPs leads to a significant alteration in the molecules displayed on the pneumococcal surface. The result of this remodelling is reduced encapsulation and perhaps a reduction in other pathophysiologically important surface proteins.

### Capsule shedding affects epithelial interactions

**Figure 6:**
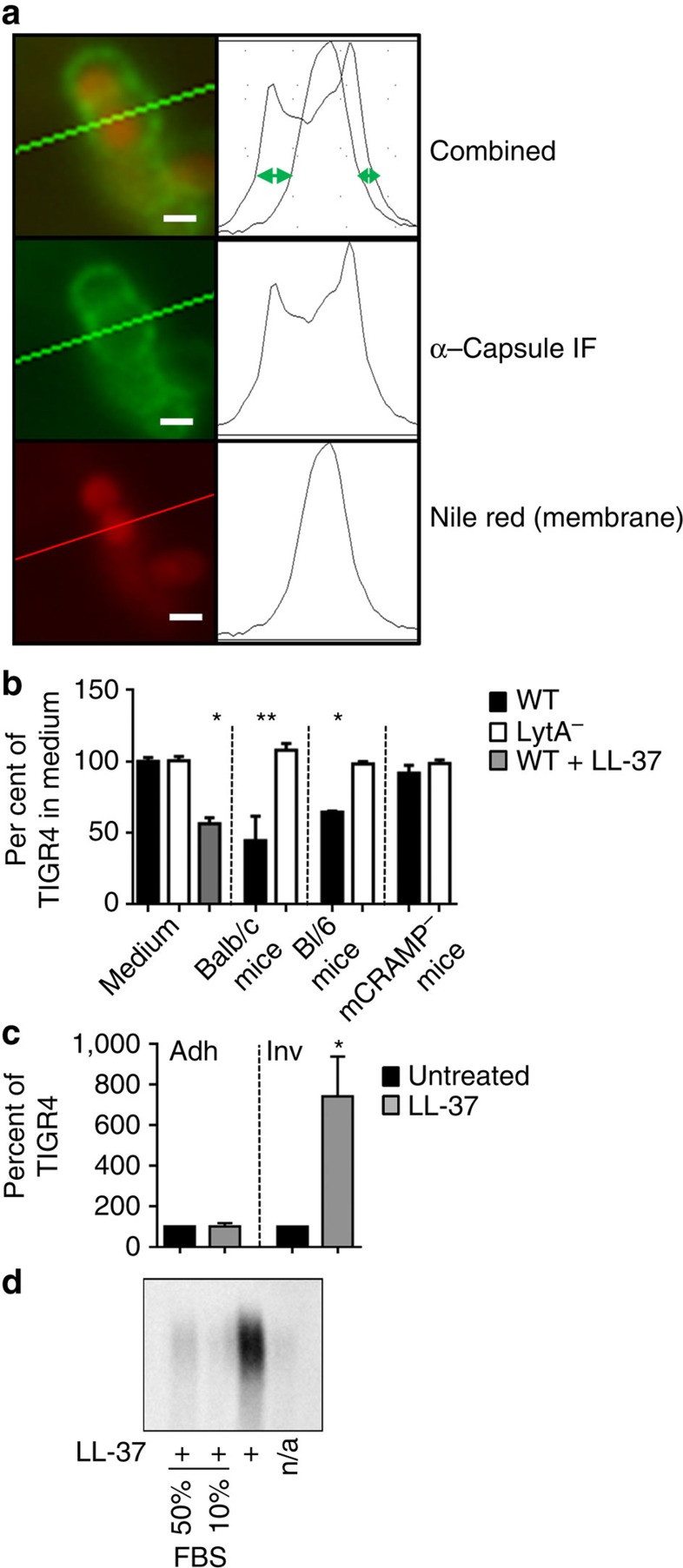
Analysis of capsule shedding *in vivo*. (**a**) Schematic representation of fluorescent microscopic measurement of encapsulation. Briefly, the diameter of the bacterial membrane (stained with Nile Red) is subtracted from the diameter of the capsule (green). The resulting distance (green arrows) is directly proportional to the amount of surface bound capsule. Scale bar, 1 μm. (**b**) Encapsulation of TIGR4 (WT) and the *lytA* mutant (LytA^−^) was measured on bacteria recovered from BAL fluid and compared with encapsulation in C+Y (medium) or after one mass doubling in 8 μg ml^−1^ LL-37 (WT+LL-37). Either Balb/c mice (WT, *n*=3 per experiment), or the cathelicidin-deficient mutant (mCRAMP^−^, *n*=3) and its WT parental control (Bl/6, *n*=3) mouse strains were infected intratracheally. After 3 h, BALs were performed and samples were subjected to analysis by fluorescent microscopy. The results are expressed as per cent encapsulation of the TIGR4 strain in medium (mean+s.e.m. from three independent experiments). *P>0.001; **P=0.0278. Unpaired *t*-test with Welch's correction. (**c**) WT (TIGR4) pneumococci grown in with or without 8 μg ml^−1^ LL-37 for one mass doubling (LL-37) were incubated with A549 epithelial cells and assayed for either adherence (adh) or invasion (inv) (see Methods). The results are mean+s.e.m. from three independent experiments. *P=0.0307. unpaired *t*-test with Welch's correction. (**d**) TIGR4 was utilized in a capsule shedding assay with 4 μg ml^−1^ LL-37. FBS added at the same time as LL-37 at the indicated concentration. The supernatant fractions were then analysed by capsule blot.

It is known that bacteria are less encapsulated on the mucosa, thereby revealing surface molecules, such as choline decorated teichoic acids, involved in bacterial attachment and invasion of the host. When pneumococci were exposed to LL-37 and then allowed to interact with a human lung epithelial cell line, we found that invasion was increased 7–8-fold (Fig. 6c). Thus, capsule shedding significantly enhanced the interaction of pneumococci with host epithelial cells, a prerequisite for invasive disease. Once past the mucosal surface, pneumococci in blood enhance encapsulation to prevent complement and antibody-mediated killing by phagocytes. CAMPs are relatively inactive in blood because of sequestration by serum proteins that prevents their lytic activity on host cells^28^. Indeed, inclusion of serum even at low levels (10% v/v) prevented capsule shedding in our standard assay (Fig. 6d). Perhaps during invasive disease, when pneumococci circulate in the blood, serum proteins inactivate residual CAMPs acquired at the epithelium and allows for the retention of capsule on the bacterial cell surface.

Tissue-specific alteration of encapsulation also occurs during the process of colony morphotype variation^29^. Phenotypic variation between opaque and transparent morphotypes is thought to involve underlying genetic changes and results in the emergence of a few colonies in a population that have altered expression of surface capsule and other phase variable proteins^30^,^31^. When tested, growth in liquid or solidified medium supplemented with LL-37 did not alter the ratio of opaque to transparent colonies (Supplementary Fig. 4) suggesting that phase transitions are not greatly influenced by CAMPs. Taken as a whole, these data indicate that rapid transition of a large portion of a population of pneumococci from encapsulated to unencapsulated observed at the epithelium is driven by the capsule shedding response.

### Capsule shedding during acute infection

Several studies have shown that pneumococci alter encapsulation or capsule production qualitatively in different tissues^2^,^32^,^33^. To test if LytA-dependent capsule shedding influenced encapsulation during infection, we first developed a quantitative method for measuring encapsulation in complex samples from the host. Previous microscopic strategies of fluorescent dextran exclusion and electron microscopy were found to be unsuitable for analysis of populations of bacteria in complex samples, so we devised a fluorescence microscopy assay to measure capsule as summarized in Fig. 6a. Using this technique, microscopic measurement from *in vitro* cultures grown in 8 μg ml^−1^ LL-37 showed good agreement of surface bound capsule with capsule blots under the same conditions (Fig. 6b medium, Fig. 2c left). When WT and LytA-deficient strains infected WT BALB/c mice in a model of acute pneumonia, WT pneumococci lost 60% of their encapsulation at 3 h, while the LytA mutant strain remained fully encapsulated (Fig. 6b); in Bl/6 mice, 40% of capsule was lost (Fig. 6b). This shows that at an early time point during acute infection, the capsule-shedding response driven by LytA is the main pathway used by pneumococci to reduce encapsulation. Mice do not produce LL-37, but have an analogous cathelicidin-derived CAMP named mCRAMP, which is thought to be functionally similar to LL-37. In mice lacking mCRAMP, it was apparent that WT pneumococci did not respond during acute lung infection by losing capsule, retaining 95% encapsulation (Fig. 6b). Taken together these data indicate that capsule shedding in response to cathelicidin-derived CAMPs is responsible for the majority of capsule loss observed as pneumococci move from site to site during acute lung infection.

## Discussion

The majority of antibacterial vaccines currently in use rely on capsular polysaccharides as a main immunogen (that is, pneumococcus, meningococcus and *Haemophilus*)^34^,^35^. The finding that one of the major roles of LytA is to drive capsule shedding has profound implications for current treatments and vaccine strategies. Shedding removes the major imunogen from the bacterial surface and as such may help pathogens evade anti-capsular immune responses. In fact, the efficacy of pneumococcal vaccines in preventing hospitalization correlates with the encapsulation of bacteria recovered from those tissues; pneumonia and otitis, diseases of the epithelium, where the pneumococcus is less encapsulated, are prevented significantly less well by capsular vaccines compared with bacteremia and meningitis^36^. In terms of bacterial physiology, the use of controlled cell wall clipping to remove capsule means that chemically diverse capsule serotypes can be swapped into a strain without the need for co-evolution of serotype-specific removal strategies. In the case of the pneumococcus, within a few years of introduction of capsule-specific vaccines, the serotypes included in the vaccine formulations have been replaced in the population by non-vaccine capsule types. Recently it has become clear through genomic analysis that rather than new virulent strains appearing to take the place of vaccine type strains, some virulent subtypes have specifically exchanged the capsule biosynthesis locus. These capsule switch mutants retain important virulence determinants and differ largely from their parent strains in capsule chemistry^37^. Therefore, the onus of diversifying the capsule locus to evade vaccines is alleviated partially by the ability of LytA to remove any capsule type acquired through recombination. The caveat is that this universal removal strategy is also a Trojan horse that maintains the same protein used by cell wall-active antibiotics to kill the bacteria.

The survival advantage of capsule removal provides an attractive model to explain why the potentially suicidal LytA is maintained in the genome of virulent isolates of pneumococci. The use of constant penicillin prophylaxis in high-risk populations would be expected to place the spatially restricted regulatory strategies controlling autolytic LytA activity under evolutionary pressure. In classical studies, antibiotic exposure has been found to result either in resistance, where the drug fails to bind to cell wall synthesis targets, or in tolerance, where the control of triggering of LytA is altered; in neither case is *lytA* ever lost^38^. When we tested a highly penicillin tolerant strain of pneumococci that harbours LytA but does not activate it in response to penicillin, we observed conservation of capsule shedding (Supplementary Fig. 3). Together this suggests that in response to antibiotics, pneumococcal evolution will result mainly in mutations altering LytA activation and control at the growth zone rather than complete loss of LytA function. It appears that loss of LytA leading to increased CAMP sensitivity and loss of capsule shedding is not well tolerated in pneumococci colonizing immune competent hosts.

Autolysis is a curious suicidal characteristic of major bacterial pathogens of children. LytA null mutants are perfectly viable and are even able to colonize the nasopharynx in some models^39^. The lytic degradation of cell wall has several impacts on the pneumococcal life cycle including release of inflammatory cell wall components altering the immune response and release of extracellular DNA, which is thought to be important in competence and biofilm formation^40^,^41^,^42^,^43^. However, in these processes the end result for LytA activation is still bacterial death; thus, the advantage of maintaining this high-risk property has been a long-standing conundrum in bacteriology. Even intense selective pressure by antibiotics has driven the emergence of downregulation of LytA activation, that is, tolerance, rather than elimination of the enzyme^44^. In fact, most penicillin-resistant isolates are tolerant but preserve *lytA* (refs 45, 46, 47). The majority of autolysin on a pneumococcal surface does not participate in lysis by antibiotics or induction of stationary phase lysis by quorum sensing as LytA is produced throughout growth, but activated only under certain circumstances^48^. Our work has identified a new physiological function for the majority of pneumococcal surface autolysin. LytA drives rapid capsule shedding in response to encountering CAMPs in the initial phases of infection of the host. This response increases bacterial resistance to these microbicidal innate defense molecules and increases the invasiveness of bacteria at the epithelium. This supports the hypothesis that pneumococci have a pre-programmed machinery to rapidly modulate the amount of surface capsule in response to host-defense signals, such that bearing less capsule while on the CAMP-rich mucosal surface promotes bacterial survival together with attachment and invasion, while replenishing of capsule in the blood protects bacteria from phagocytosis.

## Methods

### Media and growth conditions

*S. pneumoniae* strains (Supplementary Table 1) were routinely grown on tryptic soy agar (EMD Chemicals, NJ) supplemented with 3% sterile sheep blood or in liquid culture in semisynthetic casein liquid medium supplemented with 0.5% yeast extract (C+Y) (ref. 49) at 37 °C in 5% CO2 unless otherwise noted. Erythromycin was added at 1 μg ml^−1^ and kanamycin at 300 μg ml^−1^ for appropriate cultures. The antimicrobial peptides LL-37 (synthesized by St Jude Children's Research Hospital Hartwell Center), LL-37 5-TAMRA (Innovagen, cat SP-5259-1), Polymyxin B (Sigma, cat 92283), Nisin (Sigma, cat N5764) or β-defensin 3 (Anaspec, cat AS-60741) were resuspended in cell culture-grade water (Sigma) before use. Cell culture experiments utilized F12K medium (ATCC) supplemented with 10% fetal bovine serum (FBS) (ATCC). Optical density for growth curves was measured at *λ* 620 nm in 96 well plate format on a SpectraMax 340 microplate reader (Molecular Devices) and in culture tube format on a Turner Model 340 Spectrophotometer.

### Construction of mutant strains and plasmids

Mutants of *S. pneumoniae* were constructed using PCR-based overlap extension^50^, which created a deletion allele marked with *ermB,* using primers listed in Supplementary Table 2. Final PCR constructs were purified after agarose gel electrophoresis using QIAquick Gel Extraction kit (Qiagen cat # 28706) and transformed into the pneumococcus using competence stimulating peptide 1 (for D39 and derivatives), competence stimulating peptide 2 (for TIGR4 and derivatives) or a mixture of both for other strains. To confirm allelic replacement of target genes genomic DNA from recovered mutants was screened by PCR. Mutants of *lytA* in the serotype 3 strain A66.1 and the serotype 6A strain 6A4 were generated through transformation of the PCR amplified Δ*lytA*::*ermB* mutant allele from CKB410. To construct pLytA and its derivatives the *lytA* gene was amplified from TIGR4 genomic DNA using primers CK613 and CK614, then digested with XhoI and BamHI and ligated into the XhoI/BamHI sites of the streptococcal shuttle vector pABG5 (ref. 51) (gift from Michael Caparon, Washington University in St Louis) yielding pCK540. Purified pCK540 was then used as the template for PCR with the 5′phosphorylated primers CK639 and CK640 and the resulting PCR product was self-ligated yielding pLytA (pABG5:*lytA*-6xHIS, pCK584). To mutate active-site residues, pLytA was used as a template for PCR with the 5′ phosphorylated primers CK648 and CK649 and the resulting PCR product was self-ligated yielding pLytA^−^ (pABG5:*lytA*-6xHIS[H147A, D149A], pCK624). The fidelity of each plasmid construct was confirmed by sequencing of the insert portion of the resulting vector.

### Purification of LytA

Full-length recombinant LytA was purified from *E. coli* using constructs and methods found in ref. 52. A version of LytA with the predicted choline-binding domains deleted was was amplified from TIGR4 genomic DNA using primers LytANde and LytABam. The PCR product was digested with NdeI and BamHI and ligated into prepared pET28a vector. Clones were sequenced and transformed into *E. coli* BL21(DE3) competent cells (Novagen cat# 69450). Overnight cultures were induced with 1 mM IPTG for 3 h at 37 °C. Recombinant protein was purified using Ni-NTA resin (Qiagen) and dialysed overnight at 4 °C in PBS.

### Western blotting

Unless otherwise specified, logarithmic cultures of pneumococcal strains grown in C+Y with appropriate antibiotics were harvested at OD 0.4, pelleted by centrifugation, washed with PBS, pelleted then resuspended in dPBS with 1 × complete mini protease inhibitor (Roche). The cells were then lysed by two rounds of mechanical disruption using 0.2 mm silica beads in a Fast Prep machine (MP Biomedicals) using the following settings: time: 40 s speed: 6.5 m s^−1^. Samples were then boiled for 5 min, and insoluble material cleared by centrifugation. Total protein concentration was determined by BCA assay (Pierce-Thermo) and equal amounts of protein per sample were electrophoresed on 4–12% NuPAGE Bis-Tris gels using manufacturers recommended conditions (Life Technologies). After transfer to polyvinylidene difluoride membrane blots were probed with LytA polyclonal antiserum (1:5,000 dilution)^53^, pilus polyclonal antiserum (1:5,000 dilution)^54^ or CbpA polyclonal antiserum (1:5,000 dilution)^55^ and horseradish peroxidase-conjugated secondary antibody (1:10,000 dilution) (Bio-Rad cat# 170–6515). Blots were imaged using a ChemiDoc MP imaging system (Bio-Rad).

### Capsule-shedding assay and capsule blotting

An amount of 1 ml of logarithmic culture of pneumococci grown in C+Y or indicated medium was harvested at OD 0.4 by centrifugation. The pellet was then washed with 1 ml buffer SMM (0.5 M Sucrose, 0.02 M MgCl_2_, 0.02 M 2-(N-Morpholino)ethanesulfonic acid (MES) pH 6.5) and resuspended in 1 ml SMM. Either LL-37 (at 4 μg ml^−1^ for standard assays, or the indicated concentration) or antibiotics at the indicated concentration were then added and the samples were incubated at 37 °C for 30 min (or indicated time). Samples were then centrifuged 5 min at >14,000*g*, and the supernatant removed and kept for analysis (supernatant fraction). The cell pellet was then resuspended in 975 μl PBS to which 25 μl of 10% (w/v) deoxycholate was added (for LytA-deficient strains 5 μg of purified LytA was added to allow for bile salt stimulated lysis) and the cells were allowed to lyse for 30 min at 37 °C (Pellet fraction). To remove significant proteinaceous cross-reactive species detected by anti-capsule antisera, before analysis both supernatant and pellet fractions were treated with 5 μl proteinase K (Sigma, cat# P4850) for 30 min at 37 °C. To properly visualize the capsule, we adapted an alternate electrophoresis and transfer method. Past methods of polyacrylamide gel electrophoresis (PAGE) resulted in <5% of capsule entering the resolving portion of the gel, and the low molecular weight species from bacterial samples that entered PAGE gels were not apparent in commercially available purified capsular polysaccharides (ATCC, serotype 4 cat 173-X, serotype 3 cat 169-X, serotype 2 cat 165-X, serotype 6A cat 271-X and serotype 19F cat 99-X). Therefore, capsule-containing samples were boiled in SDS–PAGE sample buffer and 5 μl was electrophoresed on a 0.8% agarose gel in standard 1 × Tris-Acetate running buffer and 3 μl of a 20 μg ml^−1^ solution of purified capsular polysaccharides was included as a standard in all gels. The majority of bacterial capsule and purified capsule were resolved by this method. After electrophoresis samples were transferred from the agarose gel to mixed nitrocellulose ester membranes (HATF, Millipore) via 20 × SSC capillary transfer overnight. After transfer membranes were rinsed in 6 × SSC, allowed to air dry and UV crosslinked at 150,000 mJ using a Stratagene UV crosslinker. The membrane was then blocked with PBS containing 0.1% Tween 20 and 5% non-fat milk for 1 h at room temperature and probed with commercially available serotype-specific anti-capsular antiserum (Statens Serum Institute, serotype 4 cat 16747, serotype 2 16,745, serotype 3 16,746, serotype 6A cat 16,900 and serotype 19F cat 16,911) at a 1:15,000 dilution. After washing the membrane was then visualized using secondary horseradish peroxidase-conjugated antibodies (Bio-Rad cat 170–6515) (1:30,000) and imaged on a ChemiDoc MP imager (Bio-Rad). For quantitation of relative capsule amounts blot images were analysed by densitometry using Image Lab software (Bio-Rad).

### Ethanolamine pulse

Logarithmic cultures of pneumococci growing in C+Y were harvested at OD 0.4 by centrifugation and washed three times with C+Y devoid of choline then resuspended at OD 0.2 in choline-free C+Y. Either choline (4 μg ml^−1^ final concentration) or ethanolamine (20 μg ml^−1^ final concentration) was then added to the appropriate cultures and after one mass doubling (measured by optical density) the cultures were either harvested for capsule shedding or used directly for measuring growth after addition of penicillin at the indicated concentration.

### Adhesion and invasion

The respiratory epithelial cell line A549 (ATCC cat# CCL-185) was routinely cultured in F12K medium supplemented with 10% FBS. For adhesion and invasion assays pneumococci were harvested from logarithmic culture at OD 0.4 and resuspended in cell culture medium. A total of 1 × 10^7^ pneumococci were added to each well of 12-well tissue culture plates containing A549 cells that had been treated with tumour necrosis factor α (10 ng ml^−1^) for 2 h before the assay^56^. For adherence assays, pneumococci were incubated for 1 h with cells after which wells were washed three times with sterile PBS and cells released from the plate by trypsinization. Samples and dilutions were grown overnight on TSA blood agar plates and the resulting colony forming units (CFUs) were counted as bacteria adherent to cells. For invasion assays pneumococci were incubated with A549 cells for 2 h, after which wells were washed three times with sterile PBS and penicillin (10 μg ml^−1^) and gentamicin (200 μg ml^−1^) were added to the wells for 1 h to kill bacteria that remained extracellular. Wells were again washed three times with sterile PBS and cells were released from the plate with trypsin and lysed with cold 0.025% TritonX-100 before samples were removed and plated on blood agar plates. Resulting CFUs were counted as bacteria that had invaded A549 cells.

### Acute lung infection and animal experiments.

Six-week-old female mice from Jackson labs (Balb/c, Bl/6 or mCRAMP^−/−^ with stock numbers 000651, 000664 and 017799, respectively) were inoculated with 1 × 10^7^ CFUs of exponentially harvested pneumococci intratracheally via feeding needle essentially as described^57^. After 3 h mice were killed, and lungs were lavaged with 2 ml sterile PBS. Each experimental group consisted of three mice and all experiments were carried out with three independent replicates. All experiments were done under inhaled isoflurane anaesthesia and followed protocols approved by St Jude Children's Hospital IACUC (Institutional Animal Care and Use Committee).

### Fluorescent microscopy

To measure encapsulation, either bacteria from *in vitro* growth or BAL fluid were harvested by centrifugation and resuspended in ice-cold PBS/paraformaldehyde (4%) (Electron Microscopy Sciences, cat 15710) and allowed to fix on ice for 1 h. After fixation samples were centrifuged and washed with ice-cold PBS then resuspended in room temperature PBS and incubated with anti-capsular antibody (1:100) for 30 min. Samples were then washed twice with sterile PBS, resuspended in PBS and stained with goat anti-rabbit Alexa Fluor 488F(ab')2 fragment (Life Technologies, cat A-11070) (1:200) for 30 min at room temperature in the dark. After 30 min Nile Red stain (Life Technologies, cat N-1142) (0.1 mg ml^−1^ in DMSO) was added at 1:100 (v/v) and allowed to stain for an additional 5 min at room temperature. Samples were then collected by centrifugation, washed twice and resuspended in sterile PBS. For labelled LL-37 and Mitrotracker Green FM staining, 1 ml of culture grown as for capsule-shedding assay were incubated with 4 μg ml^−1^ LL-37-TAMRA and 200 nM Mitotracker Green FM (Life Technologies, cat M7514) for 15 min at room temperature. Samples were then collected by centrifugation, washed with 1 ml buffer SMM and resuspended in sterile PBS. Samples were mounted for imaging in 1% (w/v, final concentration) low melting temperature agarose, imaged on a Nikon Eclipse TE300 microscope equipped with Sola Light Engine (Lumincor) and Retiga 2000R camera (Qimaging), using appropriate fluorescent filters and NIS-elements (Nikon) software. After image acquisition fluorescent channels were over-layered and fluorescent intensity across a section was measured using ImageJ software. The distance (in pixels) measured for Nile red stain (membrane) was subtracted from the distance measured for Alexa Fluor 488 (capsule). For bacteria from *in vitro* cultures a minimum of 100 bacteria per condition per replicate were measured, and for BAL samples a minimum of 50 bacteria per mouse were measured.

### Statistics

Differences in the mean between experimental groups were tested for significance using unpaired *t*-test and Prism 6.0 (GraphPad) software and the null hypothesis was rejected when *P*<0.05.

## Additional information

**How to cite this article:** Kietzman, C. C. *et al.* Dynamic capsule restructuring by the main pneumococcal autolysin LytA in response to the epithelium. *Nat. Commun.* 7:10859 doi: 10.1038/ncomms10859 (2016).

## Supplementary Material

Supplementary Information Supplementary Figures 1-6, Supplementary Tables 1-3 and Supplementary References
