# Supplementary material for: Dynamic capsule restructuring by the main pneumococcal autolysin LytA in response to the epithelium
Source: Nat Commun. 2016 Feb 29;7:10859. doi: 10.1038/ncomms10859 (PMC4773454; doi:10.1038/ncomms10859)
Supplement: Supplementary Information — Supplementary Figures 1-6, Supplementary Tables 1-3 and Supplementary References [file ncomms10859-s1.pdf]

Supplementary figure 1

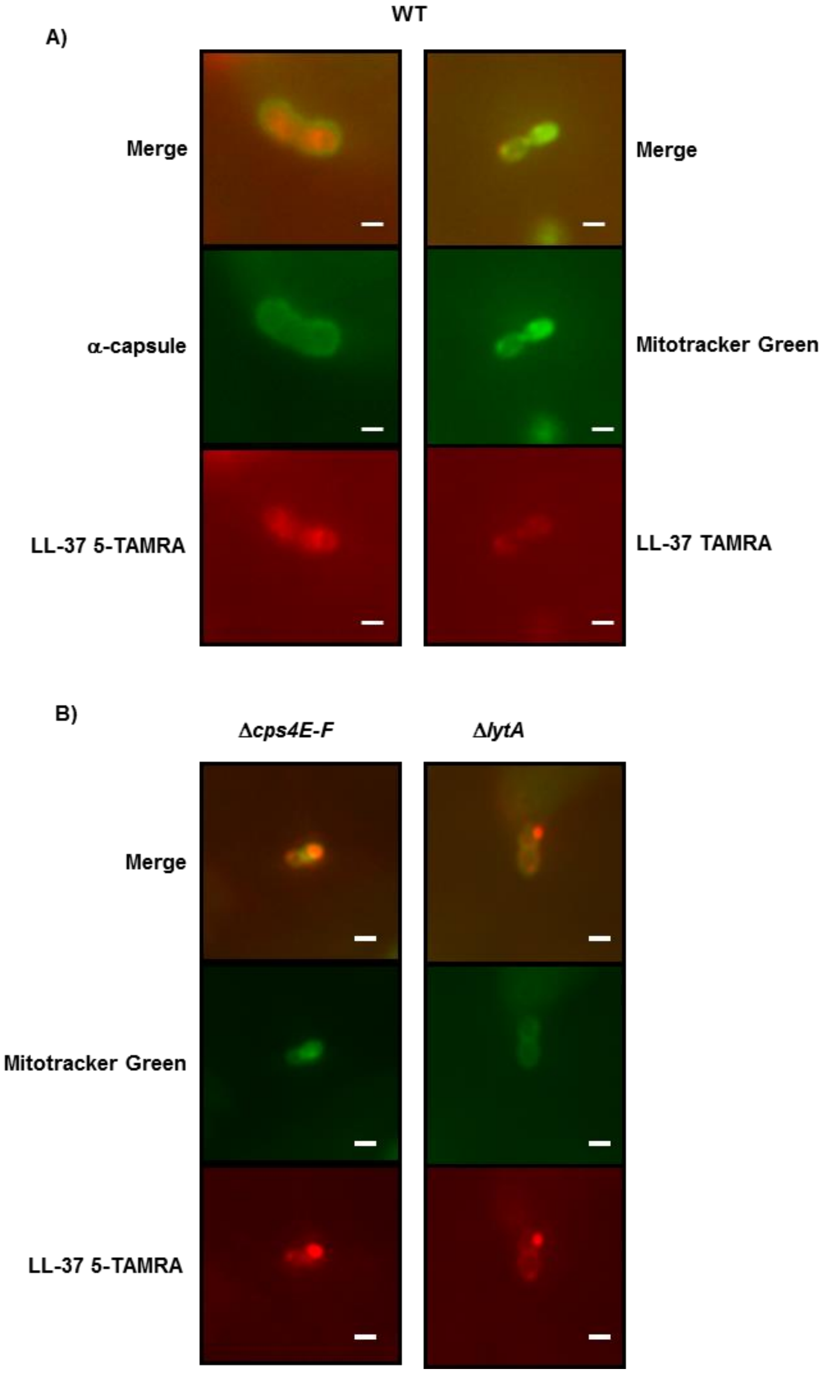

Supplementary Figure 1: Localization of LL-37. A) Wild Type TIGR4 (WT) bacteria were grown as for a shedding assay. Samples were then split and co-stained with LL-37 conjugated to a fluorescent dye (LL-37 5-TAMRA), and either anti-capsule immune-staining (α-capsule) or the membrane dye MitoTracker Green FM (Mitotracker Green). B) The mutant pneumococcal TIGR4 derivative strains Δ*cps4E-F* (unencapsulated) or ΔlytA ($LytA^-$ strain) were grown as in A), and then co-stained with LL-37 5-TAMRA and MitoTracker Green FM. All experiments were repeated three times and representative micrographs from one experiment are presented. Scale bar = 1 μm.

Supplementary Figure 2

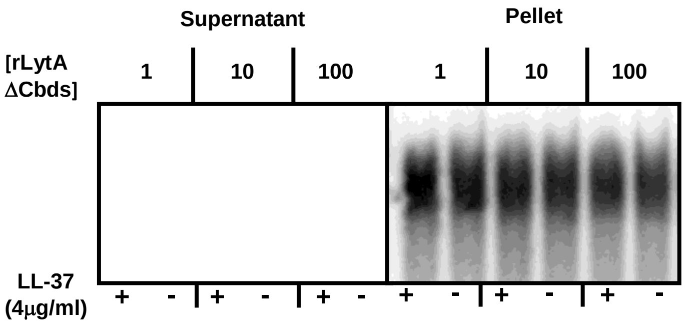

Supplementary Figure 2: Addition of LytA without choline binding domains to shedding assay. The *lytA* mutant was subjected to a capsule shedding assay, and recombinant LytA protein with choline binding domains deleted (rLytA ΔCbds) was added at the indicated concentration to the assay. LL-37 was added to the indicated cultures at 4 μg/ml. The supernatant and pellet fractions were then analyzed by capsule blot.

Supplementary Figure 3

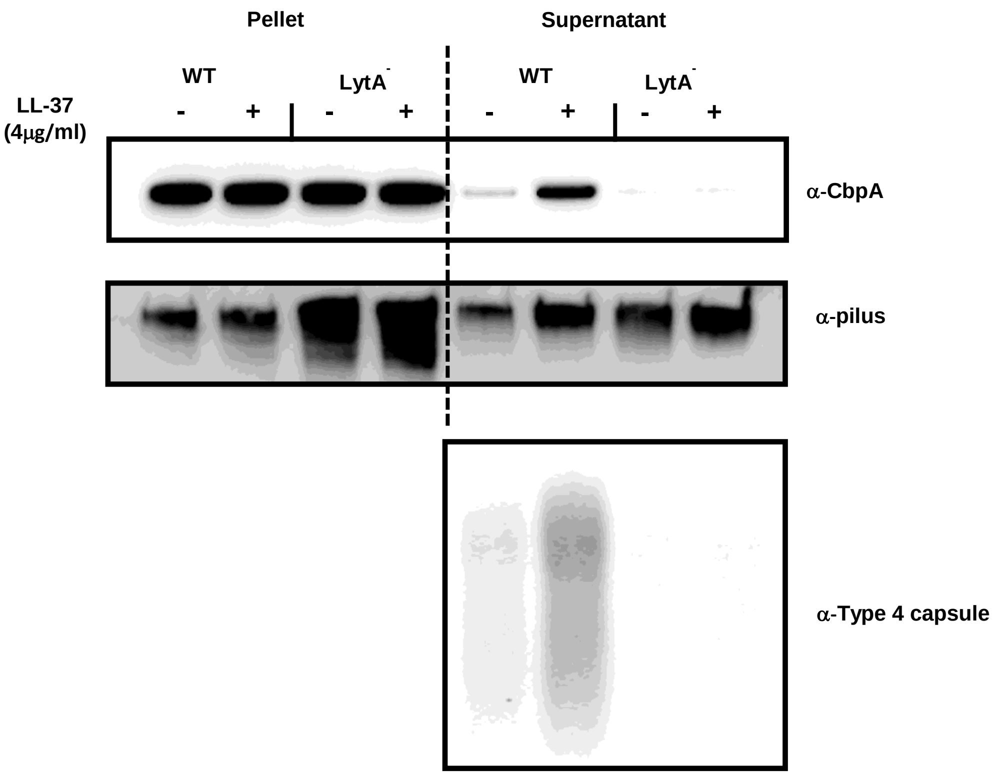

Supplementary Figure 3: Localization of cell wall attached proteins after LL-37 exposure. Either TIGR4 (WT) or the LytA- mutant (LytA-) were grown and processed for a capsule shedding assay. After separating pellet and supernatant fractions, samples were taken and analyzed by Western blot using serum against CbpA (α-CbpA), pilus (α-pilus), or capsule blot (α-Type 4 capsule). The experiment was replicated 3 times and the blots presented are from on representative experiment.

Supplementary Figure 4

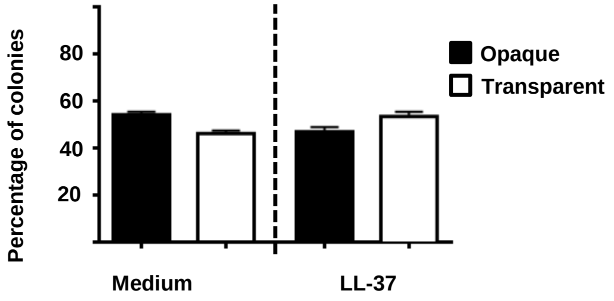

Supplementary Figure 4: Effect of LL-37 exposure on colony morphology. Wild type TIGR4 was grown in medium supplemented with LL-37 (4µg/ml) or un-supplemented (Medium) as indicated then plated on solidified agar medium. Colony morphology was assessed by observation under dissecting microscope with oblique illumination. The results are percentage of colonies displaying either opaque or transparent morphology mean and standard error from 3 independent experiments.

Supplementary Figure 5

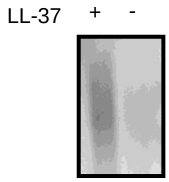

Supplementary Figure 5: Capsular shedding of a penicillin resistant strain. The highly penicillin resistant strain South Africa 8249 was subjected to a standard capsule shedding assay with 4µg/ml LL-37. The supernatant fraction was then analyzed by capsule blot.

Supplementary Figure 6

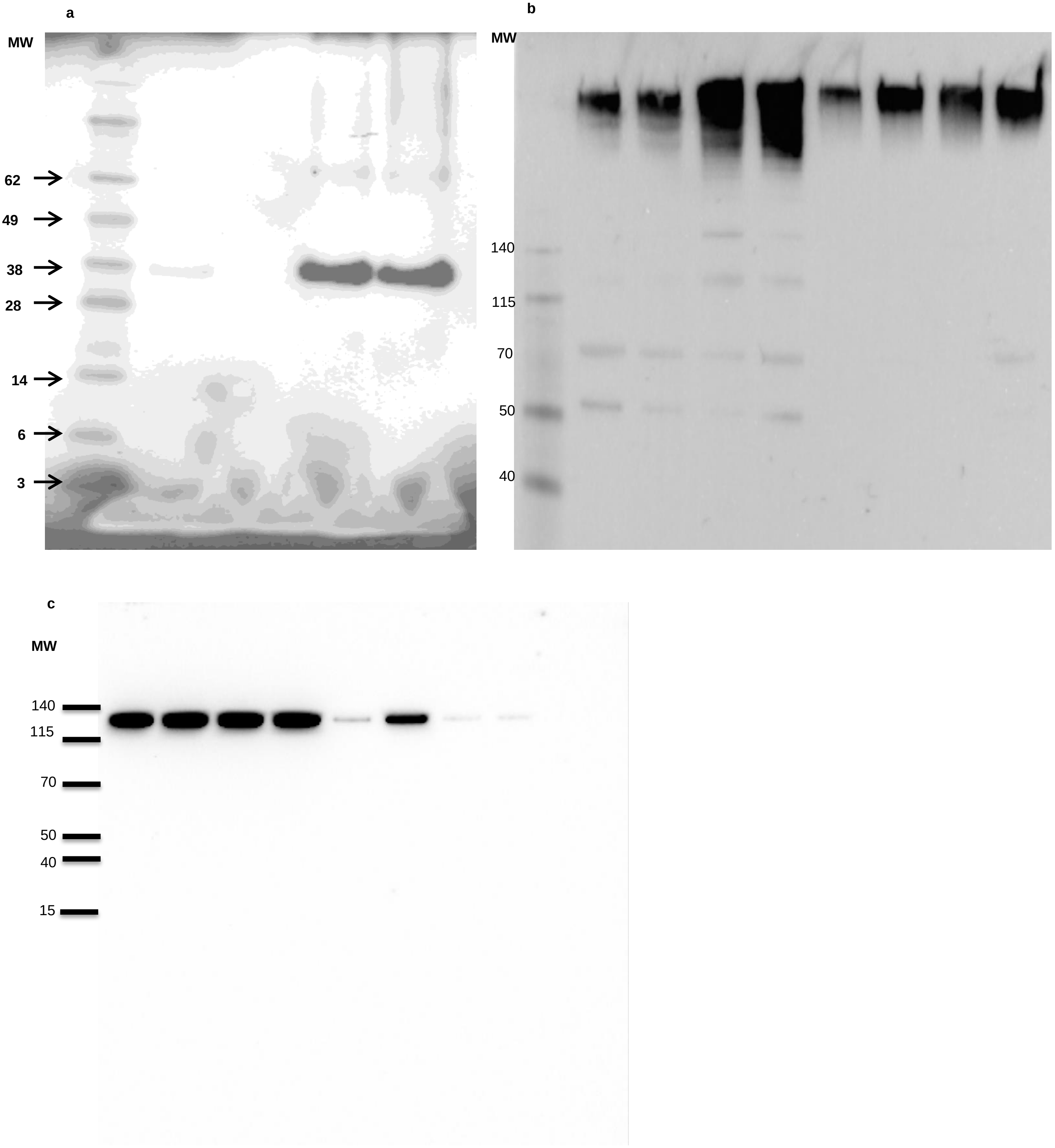

Supplementary Figure 6: Full length western blots. Full length western blots with molecular weight marker sizes indicated (MW column) in kilodaltons. Full length blot from figure 3b a). Full length blot from Supplementary figure 3 pilus b) and CbpA c).

<u>Supplementary Table 1: *Streptococcus pneumoniae* Strains used in this study</u>

Strain	Relevant Genotype	Source
TIGR4 (CKB001)	Wild type (serotype 4)	TIGR.org
LytA- (CKB410)	TIGR4 ΔSP_1937::*ermB*	This study
LytA- empty vector (CKB629)	TIGR4 ΔSP_1937::*ermB* pABG5	This study
LytA- pLytA (CKB621)	TIGR4 ΔSP_1937::*ermB* pABG5:*lytA*-6xHIS	This study
LytA- pLytA- (CKB627)	TIGR4 ΔSP_1937::*ermB pABG5:lytA*-6xHIS (H147A,D149A)	This study
TIGR4 unencapsulated (CKB424)	TIGR4 ΔSP_0360-361::*ermB*	This study
TIGR4 empty vector (CKB596)	Wild type pABG5	This study
A66.1	Wild type (serotype 3)	Perkin Elmer, cat 119247
A66.1 LytA- (CKB524)	Δ*lytA*::*ermB*	This study
6A4 (CKB502)	Wild type (serotype 6A)	[1]
6A4 LytA- (CKB512)	Δ*lytA*::*ermB*	This study
South Africa 8249	Wild type, Penicillin tolerant (serotype 19F)	[2]
D39	Wild type (serotype 2)	NCTC 7466

Supplementary Table 2: Primers Used in this Study

Purpose	Primer Name	Sequence
deletion of *lytA* (SP_1937)	CK330	CTCTTGGAGCAAGGTTTGGC
	CK505	GTTTGCTTCTAAGTCTTATTTCCCACATTAATTTCCATATTCTAC
	CK506	GAGTCGCTTTTGTAAATTTGGCCAGATGGCTTGATTACAG
	CK333	GAAAGCATCTAGTAACAGTTCC
deletion of *cps4E-4F* (SP_0360-0361)	CK351	GGAGTTCATTAAGAAGGCAG
	CK520	GTTTGCTTCTAAGTCTTATTTCCCATTCTATTTCCATTTGAC
	CK521	GAGTCGCTTTTGTAAATTTGGGAGAGGGAAAGTAAGAAAG
	CK522	CCATGAAGTTATCACAATTC
Cloning of *lytA*	CK613	CCCCTCGAGCATTTCCTCTAGTGAAAATCG
	CK614	CCCGGATCCGAGCATAACTTTCTAGTTTGC
Addition of c-terminal 6x-His tag to *lytA*	CK639	GCTGCTCATCACCATCACCATCACTAATAATAATGGAATGTCTT TCAAATC
	CK640	TTTTACTGTAATCAAGCCATC
mutagenesis of His147 and Asp149 in *lytA*	CK648	GTCTGAGTGGTTGTTTGGTTG
	CK649	GCAGTTGCACCTTATCCATATCTTGC
Cloning of *lytA* with choline binding domains deleted	LytANde	CGCGCGCATATGGAAATTAATGTGAG
	LytABam	CGCGCGGGATCCTTAGCCGTTCTCAATATC

Supplementary Table 3: Proteins tested but not involved in capsule shedding

Locus Tag	Gene Name	Description
SP_0369	*pbp1A*	Penicillin binding protein, bifunctional transglycosyltransferase/transpeptidase
SP_1027		Unknown
SP_0368		endo-alpha-N-acetylgalactosaminidase, cell wall localized
SP_1573	*lytC*	lysozyme, choline binding
SP_0965	*lytB*	endo-beta-N-acetylglucosaminidase
SP_0648	*bgaA*	beta-galactosidase, cell wall localized

Supplementary References

1. Lizcano, A., et al., *Early biofilm formation on microtiter plates is not correlated with the invasive disease potential of Streptococcus pneumoniae.* Microb Pathog, 2010. **48**(3-4): p. 124-130.
2. Zighelboim, S. and A. Tomasz, *Multiple antibiotic resistance in South African strains of Streptococcus pneumoniae: mechanism of resistance to beta-lactam antibiotics.* Rev Infect Dis, 1981. **3**(2): p. 267-76.
